# Involvement of GLUT1 and GLUT3 in the growth of canine melanoma cells

**DOI:** 10.1371/journal.pone.0243859

**Published:** 2021-02-04

**Authors:** Yoko Suwabe, Rei Nakano, Shinichi Namba, Naoya Yachiku, Manami Kuji, Mana Sugimura, Nanako Kitanaka, Taku Kitanaka, Tadayoshi Konno, Hiroshi Sugiya, Tomohiro Nakayama

**Affiliations:** 1 Laboratories of Veterinary Radiotherapy, Nihon University College of Bioresource Sciences, Kameino, Fujisawa, Kanagawa, Japan; 2 Laboratory for Cellular Function Conversion Technology, RIKEN Center for Integrative Medical Sciences, Suehiro-cho, Tsurumi, Yokohama, Kanagawa, Japan; 3 Laboratories of Veterinary Biochemistry, Nihon University College of Bioresource Sciences, Kameino, Fujisawa, Kanagawa, Japan; Duke University School of Medicine, UNITED STATES

## Abstract

The rate of glucose uptake dramatically increases in cancer cells even in the presence of oxygen and fully functioning mitochondria. Cancer cells produce ATP by glycolysis rather than oxidative phosphorylation under aerobic conditions, a process termed as the “Warburg effect.” In the present study, we treated canine melanoma cells with the glucose analog 2-deoxy-D-glucose (2-DG) and investigated its effect on cell growth. 2-DG attenuated cell growth in a time- and dose-dependent manner. Cell growth was also inhibited following treatment with the glucose transporter (GLUT) inhibitor WZB-117. The treatment of 2-DG and WZB-117 attenuated the glucose consumption, lactate secretion and glucose uptake of the cells. The mRNA expression of the subtypes of GLUT was examined and *GLUT1* and *GLUT3* were found to be expressed in melanoma cells. The growth, glucose consumption and lactate secretion of melanoma cells transfected with siRNAs of specific for *GLUT1* and *GLUT3* was suppressed. These findings suggest that glucose uptake via GLUT1 and GLUT3 plays a crucial role for the growth of canine melanoma cells.

## Introduction

Glucose is a main source of energy and carbon for mammalian cells. It is taken up by the cells via glucose transporters (GLUTs) and metabolized to pyruvate in the cytosol via glycolysis. In normal cells, glycolysis-derived pyruvate is predominantly imported into the mitochondrial matrix where it is oxidized to acetyl coenzyme A (CoA) by the pyruvate dehydrogenase complex. Acetyl CoA is subsequently incorporated into the tricarboxylic acid cycle and undergoes oxidative phosphorylation. However, the glucose metabolism of cancer cells is different from that of normal cells. Cancer cells show increased glucose uptake and glycolysis. Following glycolysis, most pyruvate is converted to lactate in the cytoplasm by the action of lactate dehydrogenase and is secreted. These alterations are evident even in the presence of oxygen to support mitochondrial respiration. This metabolic phenomenon is termed as aerobic glycolysis or the “Warburg effect” [1–3].

The transport of glucose across the plasma membrane is considered as a key rate-limiting step for glucose metabolism [4–7]. Two distinct transporter families are responsible for transporting glucose into the cell, namely, the sodium-coupled glucose co-transporter (SGLT) and the facilitative GLUT proteins. SGLTs are secondary active transporters that drive the transport of glucose and galactose using the sodium gradient across the cell membrane. In contrast, GLUTs are passive transporters involved in the transport of hexoses such as glucose and other substrates using either a chemical or an electrochemical gradient [4, 6].

There are 14 members of the mammalian GLUT family that are subdivided into three classes based on their protein sequences and structural similarities. Class I comprises GLUT1, GLUT2, GLUT3, GLUT4, and GLUT14, and class II is composed of GLUT5, GLUT7, GLUT9, and GLUT11. Class III transporters include GLUT6, GLUT8, GLUT10, GLUT12, and GLUT13 [6, 7]. Class I GLUT1-GLUT4 are the most studied and established members known for their role in glucose transport in various tissues and cell types [5, 6].

The upregulation in the expression of class I GLUTs has been demonstrated in different cancers [8–11], whereas class II and III GLUTs are not well studied. In human malignant melanoma, the expression of class I GLUTs such as GLUT1 and GLUT3 has been reported to be upregulated and correlated with clinical stages [12, 13]. Additionally, the expression of GLUT1 may predict hypoxia and glycolysis in the tumor tissue as well as the patient outcome [13]. These findings suggest that GLUTs may serve as promising therapeutic targets for melanoma treatment.

Oral malignant melanoma is a naturally occurring cancer in dogs [14]. The canine melanoma is, in general, aggressive, extremely metastatic, and highly associated with poor prognosis. Given its tendency to behave in a biologically aggressive manner similar to that observed in human melanoma, canine melanoma has been considered as a suitable disease model of human cancers [15–17].

In the present study, we investigated the effect of glucose metabolism on the growth of canine melanoma cells. We demonstrated that the suppression of glucose metabolism resulted in the inhibition of cell growth and that class I GLUT subtypes GLUT1 and GLUT3 were involved in the growth of canine melanoma cells.

## Materials and methods

### Materials

Canine melanoma cells (MCM-N1 cell line; 13-year-old male dog; chromosome number, 2n = 74) were purchased from DS Pharma Biomedical Co., Ltd. (Osaka, Japan). Canine melanoma cell lines (KMeC and CMec-1 [18–20]) were kindly provided by Dr. Takayuki Nakagawa (Laboratory of Veterinary Surgery, Graduate School of Agricultural and Life Sciences, The University of Tokyo). Lipofectamine 2000 and TRIzol were obtained from Life Technologies Co. (Carlsbad, CA). PrimeScript RT Master Mix and Ex Taq were supplied by TaKaRa Bio Inc. (Shiga, Japan). Rabbit monoclonal anti-GLUT1 (EPR3915) and anti-GLUT3 (EPR10508(N)) antibodies were obtained from Abcam (Cambridge, UK), and mouse monoclonal anti-mouse β-actin antibody (AC74) and GLUT1, GLUT3, and scramble small-interfering RNAs (siRNAs) were procured from Sigma-Aldrich Inc. (St Louis, MO). Horseradish peroxidase (HRP)-conjugated anti-rabbit IgG and anti-mouse IgG antibodies, ECL Western Blotting Analysis System, and ImageQuant LAS 4000 mini were supplied by GE Healthcare (Piscataway, NJ). Mini-PROTEAN TGX gel and polyvinylidene difluoride (PVDF) membranes were obtained from Bio-Rad (Hercules, CA), and Block Ace and complete mini EDTA-free protease inhibitor mixture were purchased from Roche (Mannheim, Germany). The Dulbecco’s modified Eagle medium with 1 g/L glucose (DMEM-LG), phenylmethanesulfonyl fluoride (PMSF), sodium fluoride, and 4-(2-hydroxyethyl)-1-piperazineethanesulfonic acid (HEPES) were obtained from Wako Pure Chemical Industries, Ltd. (Osaka, Japan), and 3-(4,5-dimethyl-2-thiazolyl)-2,5-diphenyl-2H-tetrazolium bromide (MTT) assay reagent, glucose assay kit-WST, lactate assay kit-WST from Dojindo (Tokyo, Japan). 2-(N-(7-Nitrobenz-2-oxa-1,3-diazol-4-yl)Amino)-2-Deoxyglucose (2-NBDG) was purchased from Peptide institute Inc (Osaka, Japan). StatMate IV was purchased from ATMS (Tokyo, Japan).

### Cell culture

Canine melanoma cells were maintained in static cultures in DMEM-LG supplemented with 10% fetal bovine serum (FBS) in an incubator with 5% CO_2_ at 37°C. The medium was replaced with fresh medium once a week [21]. After reaching 90%–95% confluency, the cells were harvested with 0.25% trypsin-ethylenediaminetetraacetic acid (EDTA) and suspended in the CELLBANKER 1 plus medium at a density of 2 × 10^6^ cells/500 μL for cryopreservation. Cell suspensions (500 μL) were placed in sterilized serum tubes, which were stored in a freezing vessel (BICELL) and cryopreserved at −80°C. Before experiments, the tubes were removed from the BICELL vessel and immersed in a water bath at 37°C. Thawed cell suspensions were transferred into centrifuge tubes containing DMEM-LG with 10% FBS and centrifuged at 300 ×*g* for 3 min. Cell pellets obtained were resuspended in DMEM-LG with 10% FBS and transferred into 75-cm^2^ culture flasks. Static culture was then carried out under the same condition as prior to cryopreservation. Cells were harvested using 0.25% trypsin-EDTA once they reached approximately 90% confluency. The collected cells were seeded at a density of 1 × 10^6^/75-cm^2^ culture flask.

### MTT assay

Cells were seeded at a density of 3,000 cells/200 μL in each well of a 96-well plate. MTT assay reagent was dissolved in phosphate-buffered saline (PBS) at a concentration of 5 mg/mL, and 20 μL of the reagent was incubated with cells for 1 h in an incubator with 5% CO_**2**_ at 37°C. Following incubation, PBS (100 μL) was added to each well. After 1 min, the supernatant was discarded and the MTT formazan crystals were dissolved in 200 μL of 0.04 M hydrochloric acid (HCl) in 2-propanol. The optimal density (O.D.) was detected by a microplate reader (Fluoroskan Ascent FL, Thermo Fisher Scientific K.K., Kanagawa, Japan) at 570-nm wavelength.

### Glucose and lactate assay

Cells were seeded at a density of 3 × 10^**5**^ cells/mL in each well of a 6-well plate. The cells were stimulated with 5 mM 2-deoxy-D-glucose (2-DG) or 60 μM WZB-117 for 72 h and culture supernatants were collected. To measure culture supernatant glucose concentrations, we used an assay kit according to the kit instructions.

### 2-NBDG uptake assay

Cells were seeded at a density of 3 × 10^**5**^ cells/mL in 35-mm glass-base dish. The cells were stimulated with 5 mM 2-deoxy-D-glucose (2-DG) or 60 μM WZB-117 for 24 h. 2-NBDG was dissolved in phosphate-buffered saline (PBS) with 10% FBS at a concentration of 50 μM, and 1 mL of the 2-NBDG reagent was incubated with the cells for 30 min in an incubator with 5% CO_**2**_ at 37°C. Following incubation, the cells were washed with PBS and fixed with 1 mL of 4% PFA in PBS to each well for 15 min. The fluorescence signal was visualized using a confocal laser scanning microscope (LSM-510; Carl Zeiss AG, Oberkochen, Germany).

### Real-Time Polymerase Chain Reaction (RT-PCR)

Total RNA was extracted from canine melanoma cells using the TRIzol^®^ reagent (Life Technologies Co.) according to the manufacturer’s instructions. RNA concentration was spectrophotometrically measured by reading the absorbance at 260 nm/280 nm. First-strand cDNA synthesis was carried out using 500 ng of the total RNA and the PrimeScript RT Master Mix (TaKaRa Bio Inc., Shiga, Japan). PCR was performed using 2 μL of first-strand cDNA in a 10-μL total reaction volume with primers specific for GLUT1, GLUT2, GLUT3, and GLUT4 (Table 1) and Ex Taq. PCR was conducted on an iCycler (Bio-Rad, Hercules, CA) as previously reported [18–29]. The thermal cycler was programmed for initial denaturation at 94°C for 2 min, followed by 25 cycles of denaturation at 94°C for 30 s, primer annealing at 55°C for 30 s, and primer extension at 72°C for 30 s. PCR products were separated using 2% agarose gel electrophoresis, stained with ethidium bromide, and visualized under UV light. The amplification of TATA box-binding protein (TBP) from the same amount of cDNA was applied as an endogenous control.

**Table 1:** Primers used for RT-PCR.

Gene Name	GenBank ID	Primer sequences	size (bp)
*SLC2A1*	NM_001159326.1	F: 5′- AGCTGCCATTGCTGTTGCTG -3′	115
(GLUT1)		R: 5′- CACGGTGAAGATGATGAAGACGTA -3′	
*SLC2A2*	XM_005639915.1	F: 5′-TGTGTGTGCCATCTTCATGTCC-3′	149
(GLUT2)		R: 5′-AGAACTCTGCCACCATGAACCA-3′	
*SLC2A3*	NM_001003308.1	F: 5′-CTTCAGATCGCGCAGCTACC-3′	118
(GLUT3)		R: 5′-TGCATCTTTGAAGATTCCTGTTGAG-3′	
*SLC2A4*	NM_001159327.1	F: 5′-GCTTCTGCAACTGGACAAGCAA-3′	178
(GLUT4)		R: 5′-AAGTCAGCCGAGATCTGGTCAA-3′	
*TBP*	XM_863452	F: 5′-ACTGTTGGTGGGTCAGCACAAG-3′	184
		R: 5′-ATGGTGTGTACGGGAGCCAAG-3′	

### Western blotting

Western blotting was performed as per methods reported previously [21–32]. The melanoma cells were lysed using a lysis buffer containing 20 mM HEPES, 1 mM PMSF, 10 mM sodium fluoride, and a complete mini EDTA-free protease inhibitor cocktail at pH 7.4. Protein concentrations were adjusted using the Bradford method [33]. Extracted proteins were boiled at 95°C for 5 min in a sodium dodecyl sulfate (SDS) buffer. Samples were loaded into separate lanes of 7.5% or 12% Mini-PROTEAN TGX gels and electrophoretically separated. Separated proteins were transferred onto PVDF membranes, treated with Block Ace for 50 min at room temperature, and incubated with primary antibodies (GLUT1 [1:1000], GLUT3 [1:1000], and β-actin [1:10000]) for 120 min at room temperature. After washing, the membranes were incubated with an HRP-conjugated anti-rabbit or anti-mouse IgG antibody (1:10000) for 90 min at room temperature. Immunoreactivity was detected using ECL Western Blotting Analysis System. Chemiluminescent signals of the membranes were measured using the ImageQuant LAS 4000 mini.

### Transfection of siRNA

The siRNA transfection was performed as per previously described methods with some modifications [24, 27–32]. Canine melanoma cells seeded at a density of 1 × 10^5^ cells/35-mm dish or 5 × 10^5^ cells/90-mm dish were transfected with Opti-MEM containing 5 μL/mL Lipofectamine 2000 and 100 nM GLUT1, GLUT3, or scramble siRNA for 6 h. After transfection, the medium was changed to DMEM-LG containing 10% FBS, and the cultures were maintained in an incubator with 5% CO_2_ at 37°C for 5 days. The siRNA sequences that were used have been shown in Table 2. The efficiency of siRNA transfection was determined by western blotting.

**Table 2:** Sequences for siRNA transfection.

Gene Name	Gene bank ID	siRNA sequences
*SLC2A1* (GLUT1)	NM_001159326.1	GCUGUCUUCUAUUACUCCA
*SLC2A3* (GLUT3)	NM_001003308.1	GCUGUUUGUUCCAUCCUUA

### Statistical analysis

The data from experiments are presented as mean ± standard error of means. Statistical analysis was performed using StatMate IV. The data from the time course study were analyzed using two-way analysis of variance, and the data from other experiments for multiple comparison and paired comparison were analyzed using one-way analysis of variance and paired t test, respectively.

## Results

### Effect of 2-deoxy-D-glucose on the growth of canine melanoma cells

**Fig 1:**
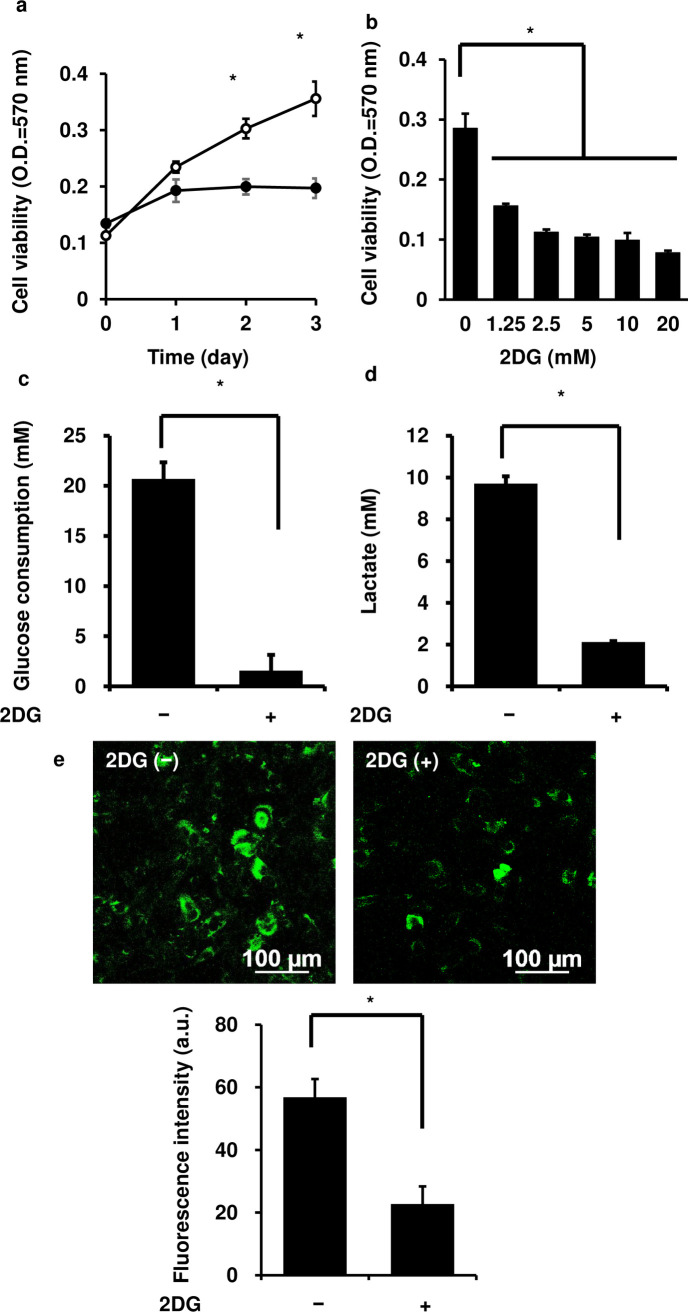
Effect of 2-deoxy-D-glucose on the growth of canine melanoma cells. (a) Time-dependent changes in the growth of canine melanoma cells with (closed circle) or without (open circle) 2-deoxy-D-glucose (2-DG) treatment. The cells were incubated with 5 mM 2-DG for 3 days, and their growth was found to be significantly attenuated. (b) Dose-dependent effect of 2-DG on the growth of canine melanoma cells. The cells were incubated with 0 to 20 mM 2-DG, which attenuated their growth in a dose-dependent manner. (c) The effect of 2-DG on glucose consumption of canine melanoma cells. The cells were incubated with 5 mM 2-DG for 3 days, and glucose consumption was found to be significantly attenuated. (d) The effect of 2-DG on lactate secretion of canine melanoma cells. The cells were incubated with 5 mM 2-DG for 3 days, and lactate secretion was found to be significantly attenuated. (e) The effect of 2-DG on glucose uptake of canine melanoma cells. The cells were incubated with 5 mM 2-DG for 3 days, and glucose uptake was found to be significantly attenuated. Results are shown as mean ± SE from three independent experiments. *P < 0.05 as compared to that on 0 day (a) or with 0 mM (b).

We first examined the contribution of glucose metabolism to the growth of canine melanoma cells using 2-deoxy-D-glucose (2-DG), a synthetic glucose analog. 2-DG is frequently used as an inhibitor of glucose metabolism, including glycolysis [34–39]. The treatment with 5 mM 2-DG for 3 days resulted in a significant decrease in the growth of cells (Fig 1A). The growth of the cells was inhibited after treatment with various concentrations of 2-DG (0 to 20 mM) for 3 days in a dose-dependent manner (Fig 1B). Glucose consumption and lactate secretion of the cells were attenuated after treatment with 5 mM 2-DG for 3 days (Fig 1C and 1D). As shown in Fig 1E, the 2-DG treatment also attenuated glucose uptake. We observed that the effect of 2-DG on the cellular viability, glucose consumption, lactate secretion and glucose uptake was conserved in several canine melanoma cell lines (S1, S2 and S5 Figs). These results suggest that melanoma cells rely on glucose metabolism for their growth.

### Effect of a GLUT inhibitor on the growth of canine melanoma cells

**Fig 2:**
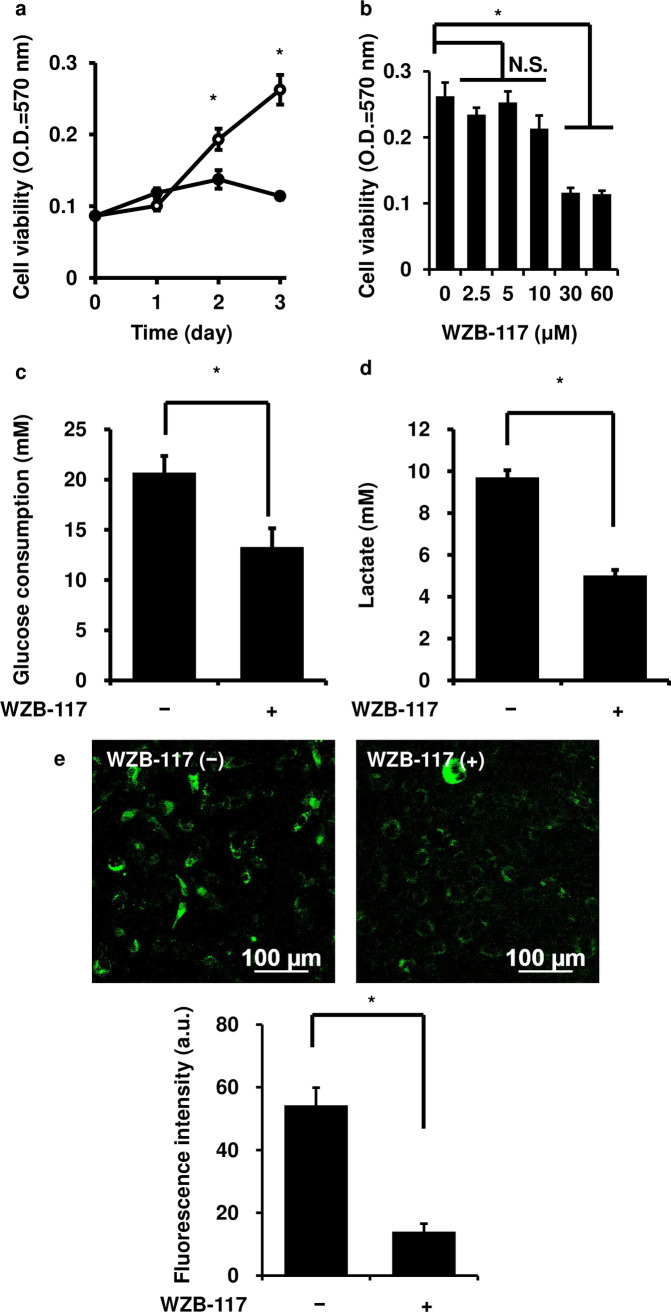
Effect of a GLUT inhibitor on the growth of canine melanoma cells. (a) Time-dependent changes in the growth of canine melanoma cells with (closed circle) or without (open circle) GLUT inhibitor WZB-117 treatment. The cells were incubated for 3 days with 60 μM WZB-117, which significantly attenuated their growth. (b) Dose-dependent effect of WZB-117 on the growth of canine melanoma cells. The cells were incubated with 0 to 60 μM WZB-117, which attenuated their growth in a dose-dependent manner. (c) The effect of WZB-117 on glucose consumption of canine melanoma cells. The cells were incubated with 60 μM WZB-117 for 3 days, and glucose consumption was found to be significantly attenuated. (d) The effect of WZB-117 on lactate secretion of canine melanoma cells. The cells were incubated with 60 μM WZB-117 for 3 days, and lactate secretion was found to be significantly attenuated. (e) The effect of WZB-117 on glucose uptake of canine melanoma cells. The cells were incubated with 60 μM WZB-117 for 3 days, and glucose uptake was found to be significantly attenuated. Results are shown as mean ± SE from three independent experiments. *P < 0.05 as compared to that on 0 day (a) or with 0 μM (b).

The production of ATP by glycolysis under aerobic conditions (Warburg effect) is important for the growth of several types of cancer cells. Glucose transport across the cell membrane via GLUTs is considered as a rate-limiting step for glycolysis. Thus, we examined the effect of the GLUT inhibitor WZB-117 on the growth of melanoma cells. The cells were incubated with 60 μM WZB-117 for 3 days that led to a significant attenuation of their growth (Fig 2A). The treatment of cells with 0 to 60 μM WZB-117 resulted in a significant decrease in their growth in a dose-dependent manner (Fig 2B). When the cells were treated with 60 μM WZB-117 for 3 days, glucose consumption and lactate secretion of the cells were significantly attenuated (Fig 2C and 2D). We also observed that the WZB-117 treatment also attenuated glucose uptake (Fig 2E). As shown in S3, S4 and S5 Figs, WZB-117 showed the significant decrease in the cellular viability, glucose consumption, lactate secretion and glucose uptake of several canine melanoma cell lines.

### Contribution of GLUT isoforms to the growth of canine melanoma cells

**Fig 3:**
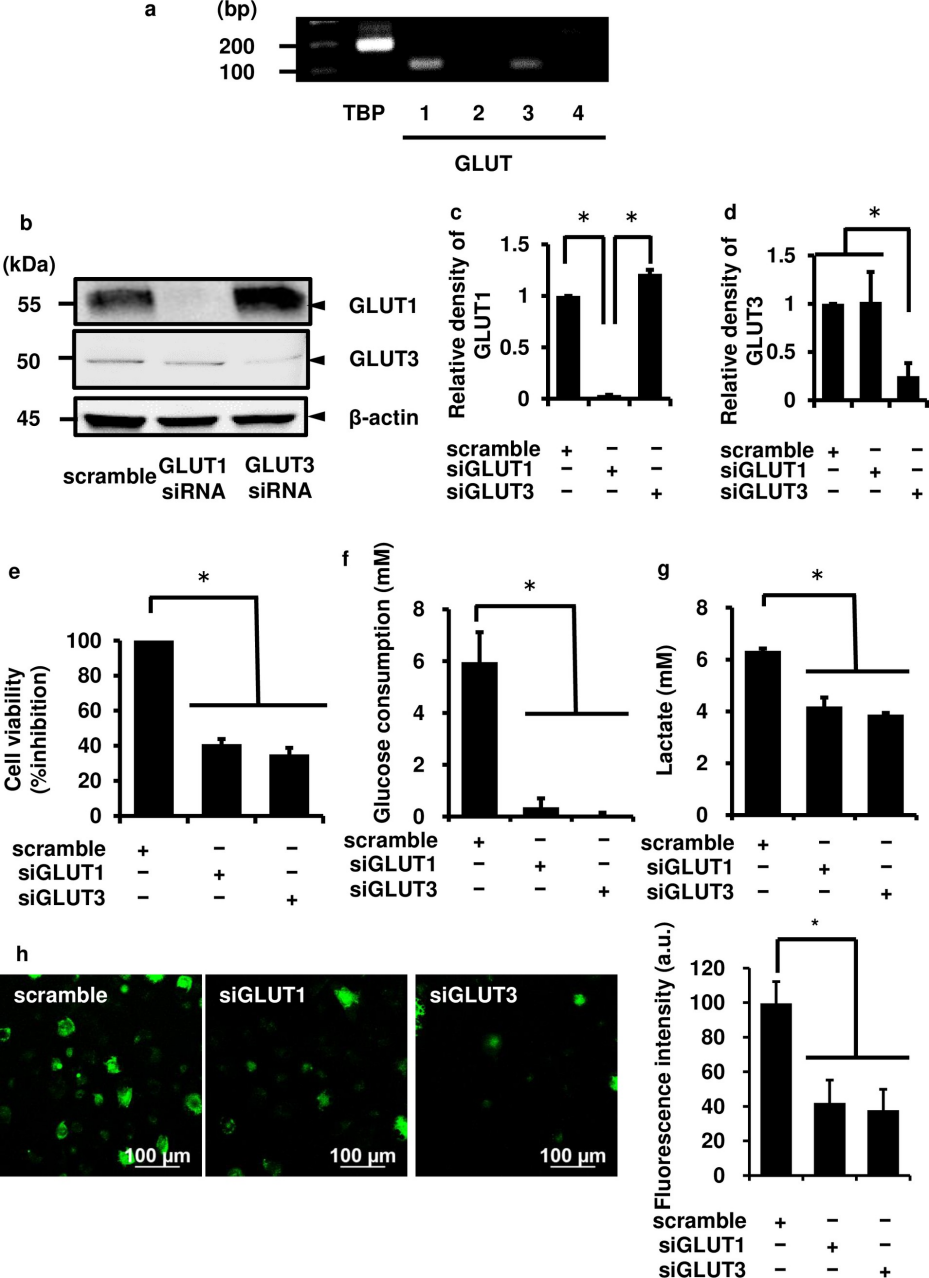
Contribution of GLUT isoforms to the growth of canine melanoma cells. (a) The mRNA expression of GLUT isoforms was detected by RT-PCR. The mRNA expression of *GLUT1* and *GLUT3*, but not *GLUT2* and *GLUT4*, was observed in canine melanoma cells. (b-d) The cells were transfected with GLUT1, GLUT3, and scrambled siRNAs and the expression of GLUT1, GLUT3, and β-actin was detected by western blotting. GLUT1 and GLUT3 siRNA transfection decreased the expression of GLUT1 and GLUT3, respectively, while scrambled siRNA transfection did not alter their expression. β-actin was used as an internal standard. Representative results (b) and relative density of GLUT1 or GLUT3 protein expression in siRNA-transfected cells as compared to those in scrambled siRNA-transfected cells (c, d) are shown. (e) The growth of canine melanoma cells was attenuated after 3 days of GLUT1 and GLUT3 siRNA transfection. (f) The glucose consumption of canine melanoma cells was attenuated after 3 days of GLUT1 and GLUT3 siRNA transfection. (g) The lactate secretion of canine melanoma cells was attenuated after 3 days of GLUT1 and GLUT3 siRNA transfection. (h) Glucose uptake was found to be significantly attenuated in the cells transfected with GLUT1 and GLUT3 siRNA. Results are presented as mean ± SE from three independent experiments. *P < 0.05.

We investigated the mRNA expression of GLUT isoforms in canine melanoma cells by RT-PCR. As shown in Fig 3A, the mRNA expression of *GLUT1* and *GLUT3*, but not that of *GLUT2* and *GLUT4*, was detected in canine melanoma cells. We observed that *GLUT1* and *GLUT3* mRNA dominantly expressed in additional canine melanoma cell lines (S6 Fig). The protein expression of GLUT1 and GLUT3 was also observed by western blotting (Fig 3B, scramble control).

To elucidate the contribution of GLUT1 and GLUT3 to the growth of canine melanoma cells, we examined the effect of the knockdown of GLUT1 and GLUT3 expression by siRNA transfection. The expression of GLUT1 and GLUT3 proteins decreased in the cells transfected with GLUT1 and GLUT3 siRNAs, respectively, but not that in those transfected with scrambled siRNA (Fig 3B–3D). The expression of β-actin used as an internal standard showed no alterations following transfection of cells with GLUT1, GLUT3, and scramble siRNAs (Fig 3B). The growth, glucose consumption, lactate secretion and glucose uptake of the cells transfected with GLUT1 and GLUT3 siRNAs were clearly attenuated (Fig 3E–3H). These results suggest that GLUT1 and GLUT3 play an important role in the growth of melanoma cells. Therefore, GLUT1 and GLUT3 may serve as promising targets for anticancer therapy of melanoma.

## Discussion

In this study, we demonstrated that 2-DG induced inhibition of the growth of canine melanoma cells. 2-DG is a glycolytic inhibitor that is taken up by cells via GLUTs as well as glucose, and is subsequently phosphorylated by hexokinase to form 2-deoxy-D-glucose-6-phosphate (2-DG-6-P). 2-DG-6-P fails to undergo further metabolism via glycolysis but accumulates and inhibits hexokinase and phosphoglucose isomerase, which are involved in the first step of glucose metabolism [40–42]. The consequences involve downstream inhibition of glycolysis and induction of oxidative stress [1, 39]. Although this effect of 2-DG is known, 2-DG is thought to interfere with *N*-linked glycosylation of proteins [35], consequently leading to endoplasmic reticulum stress and apoptosis [36]. As the depletion of glucose can lead to cancer cell death via more than one mechanism, 2-DG is considered as a potent therapeutic agent against various cancer cells [36–39]. Therefore, our observations support the notion that the anti-glycolytic strategy via depletion of glucose is promising for canine melanoma treatment.

We also demonstrated that the GLUT inhibitor WZB-117 inhibited the growth of canine melanoma cells. This observation suggests that the inhibition of GLUTs results in the suppression of cell growth by reducing glycolysis because glucose is transported across the cell membrane via GLUT in a rate-limiting step [4–7]. In a previous study, the increase in the glucose uptake rate was observed during cellular alterations and conversion to malignancy in fibroblasts infected with sarcoma viruses [43]. Upregulation in glucose transport has been considered to be associated with the overexpression of GLUTs. In human melanoma cell lines such as WM3211, Mel-Im and SbCl2, the dose-dependent reduction in glucose consumption and cell growth has been previously observed following treatment with a GLUT inhibitor [44]. Therefore, the inhibition of GLUTs could be exploited as an anticancer strategy for canine melanoma.

We found that canine melanoma cells expressed GLUT1 and GLUT3. GLUT1 and GLUT3 have high affinity (*K*m 3 and 1.4 mmol/L, respectively) for glucose that may contribute to optimization of the energy supply, thereby providing a fundamental advantage for the growth of cancer cells [45, 46]. In humans, the upregulation in the expression of GLUT1 and GLUT3 has been observed in the majority of cancers (e.g., lung, brain, breast, bladder, cervical, colorectal, esophageal, hepatocellular, ovarian, renal cell, pancreatic, and prostate cancers) and has been linked to poor survival and tumor aggressiveness [10, 47, 48]. In stage I non-small cell lung carcinoma [49], oral squamous cell carcinoma [50], breast carcinoma [51], thyroid carcinoma [52], and laryngeal carcinoma [53], the overexpression of both GLUT1 and GLUT3 genes and proteins has been reported to be associated with poor survival. Therefore, the deregulated expression of these GLUTs has been associated with malignancy [54]. Regarding human melanoma, GLUT1 and GLUT3 are expressed in malignant and benign melanocytic lesions [55]. However, GLUT1 expression was found to be downregulated in malignant melanoma in this study. On the other hand, GLUT1 and GLUT3 are considered as potentially useful markers for the differentiation of melanoma from nevi, as patients with GLUT1- and GLUT3-positive melanomas had significantly lower survival rate than those who lacked GLUT1 and GLUT3 expression in their melanomas [13, 56, 57]. Therefore, it is conceivable that the upregulation of GLUT1 and GLUT3 expression is related to the malignancy of melanoma as well as other cancers. However, the contribution of GLUT1 and GLUT3 to this biological phenomenon has been unclear in melanoma cells. The growth of GLUT1 siRNA-transfected mouse B16 melanoma cells was reduced [41]. We also demonstrated that the transfection with GLUT1 and GLUT3 siRNAs resulted in the attenuation of the growth of canine melanoma cells. The uptake of the glucose analogue 2-deoxy-2-[^**18**^F]-fluoro-D-glucose is closely related to GLUT1 and GLUT3 expression in malignant melanoma [58]. Taken together, it is likely that both GLUT1 and GLUT3 contribute to the glucose transport necessary for the growth of melanoma cells.

Silencing of GLUT1 and GLUT3 expressions by siRNA transfection was shown to enhance the apoptosis of oral squamous cell carcinoma cells and acute myeloid leukemia cells, respectively [59, 60]. Therefore, siRNA interference against GLUT1 and GLUT3 appears to be a valuable tool for the gene therapy of canine melanoma. As GLUT1 has been reported to be ubiquitously distributed in all tissues [10] and GLUT3 is predominantly expressed in neuronal tissues, placenta, testis, myocardium, and platelets in humans [10, 61–63], GLUT3 appears to be a more promising therapeutic target for the specific treatment of melanoma.

## Conclusion

As melanoma is less responsive to the existing therapeutic approach, our observations provide a new insight into the role of GLUT subtypes, GLUT1 and GLUT3, in cell growth and highlight an anti-glycolytic strategy as a promising tool for the treatment of canine melanoma.

## Supporting information

S1 Fig The effect of 2-DG on cell growth of several canine melanoma cell lines (KMeC and CMec-1). The cells were incubated with 5 mM 2-DG for 3 days, and cell growth was found to be significantly attenuated.
(PDF)

S2 Fig The effect of 2-DG on glucose consumption and lactate secretion of several canine melanoma cell lines (KMeC and CMec-1). The cells were incubated with 5mM 2-DG for 3 days, and glucose and lactate secretion were found to be significantly attenuated.
(PDF)

S3 Fig The effect of WZB-117 on cell growth of several canine melanoma cell lines (KMeC and CMec-1). The cells were incubated with 60μM WZB-117 for 3 days, and cell growth was found to be significantly attenuated.
(PDF)

S4 Fig The effect of WZB-117 on glucose consumption and lactate secretion of several canine melanoma cell lines (KMeC and CMec-1). The cells were incubated with 60 μM WZB-117 for 3 days, and glucose consumption and lactate secretion were found to be significantly attenuated.
(PDF)

S5 Fig The effect of WZB-117 on glucose consumption and lactate secretion of several canine melanoma cell lines (KMeC and CMec-1). The cells were incubated with 5 mM 2-DG or 60 μM WZB-117 for 24 h, and glucose uptake was found to be significantly attenuated.
(PDF)

S6 Fig The MRNA expression of GLUT isoforms in canine melanoma cell lines. (PDF)

S7 Fig Uncropped images for the blots shown in Fig 3. (PDF)
