# Supplementary material for: Involvement of GLUT1 and GLUT3 in the growth of canine melanoma cells
Source: PLoS One. 2021 Feb 4;16(2):e0243859. doi: 10.1371/journal.pone.0243859 (PMC7861381; doi:10.1371/journal.pone.0243859)
Supplement: S1 Fig — The cells were incubated with 5 mM 2-DG for 3 days, and cell growth was found to be significantly attenuated. (PDF) [file pone.0243859.s001.pdf]

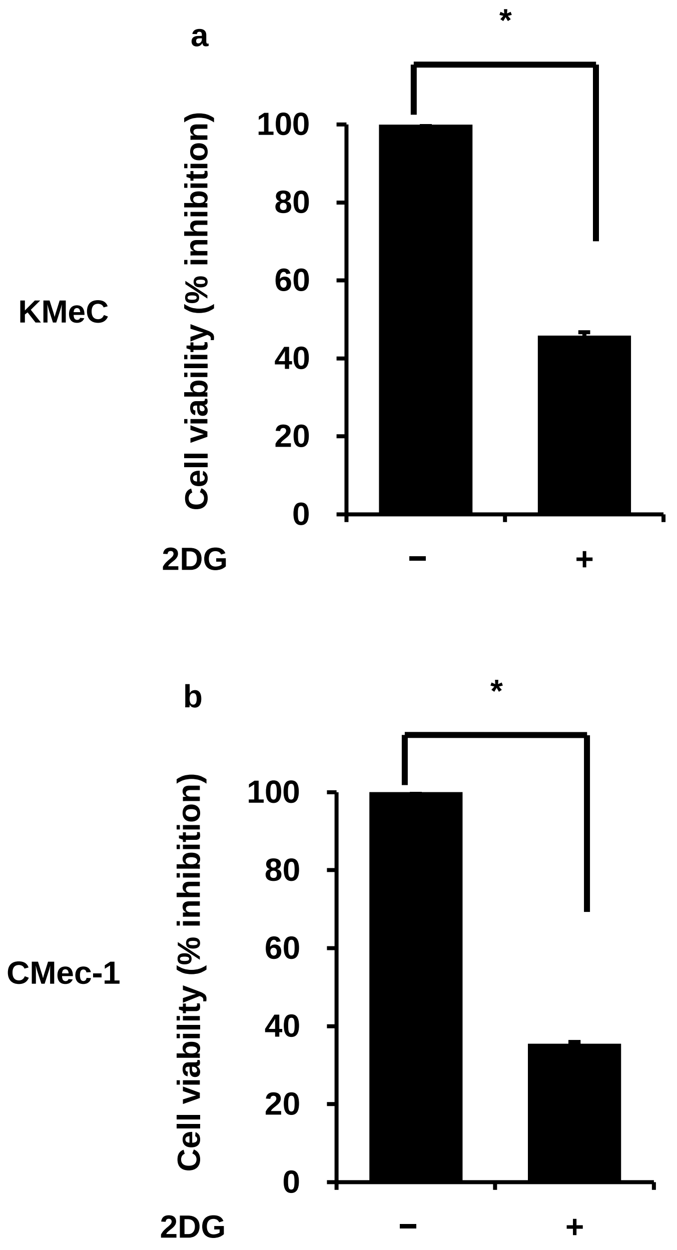

S1 Fig. The effect of 2-DG on cell growth of several canine melanoma cell lines (KMeC and CMec-1). The cells were incubated with 5 mM 2-DG for 3 days, and cell growth was found to be significantly attenuated.
