# Supplementary material for: Involvement of GLUT1 and GLUT3 in the growth of canine melanoma cells
Source: PLoS One. 2021 Feb 4;16(2):e0243859. doi: 10.1371/journal.pone.0243859 (PMC7861381; doi:10.1371/journal.pone.0243859)
Supplement: S3 Fig — The cells were incubated with 60μM WZB-117 for 3 days, and cell growth was found to be significantly attenuated. (PDF) [file pone.0243859.s003.pdf]

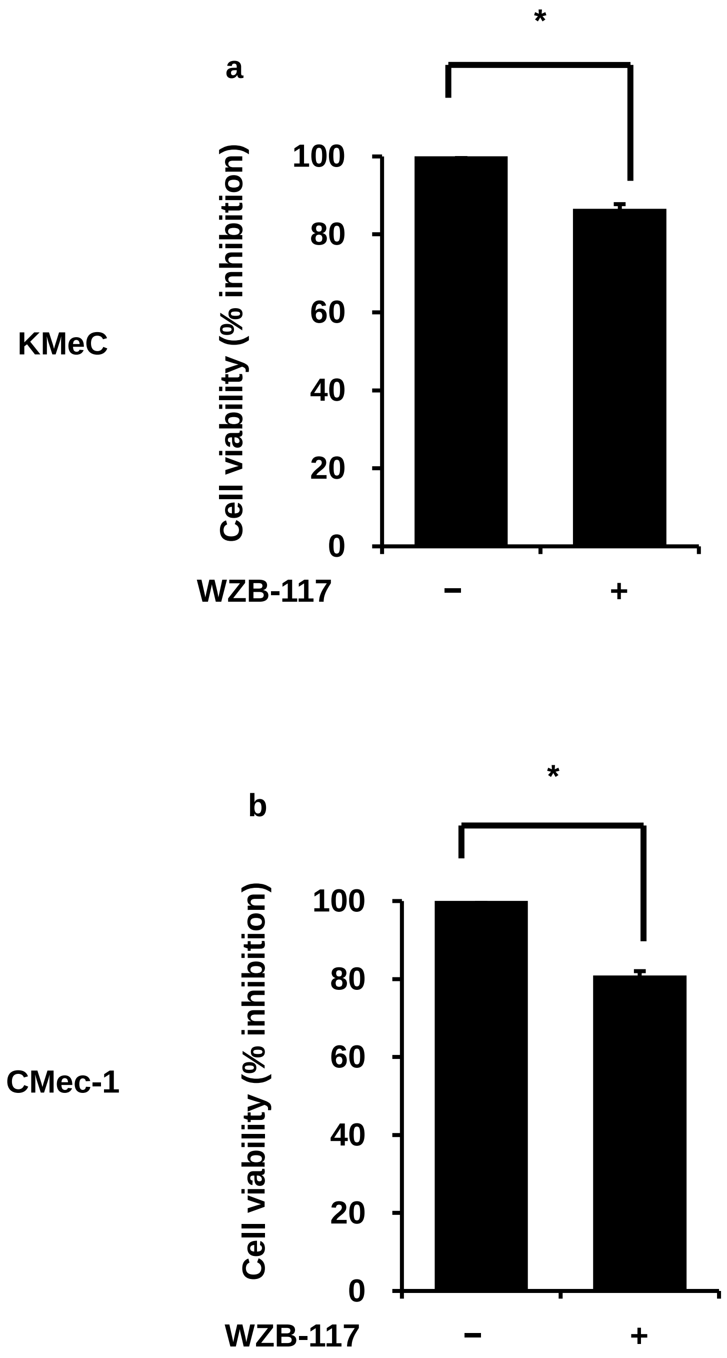

S3 Fig. The effect of WZB-117 on cell growth of several canine melanoma cell lines (KMeC and CMec-1). The cells were incubated with 60 µM WZB-117 for 3 days, and cell growth was found to be significantly attenuated.
