# Supplementary figures and images for: Involvement of GLUT1 and GLUT3 in the growth of canine melanoma cells
Source: PLoS One. 2021 Feb 4;16(2):e0243859. doi: 10.1371/journal.pone.0243859 (PMC7861381; doi:10.1371/journal.pone.0243859)

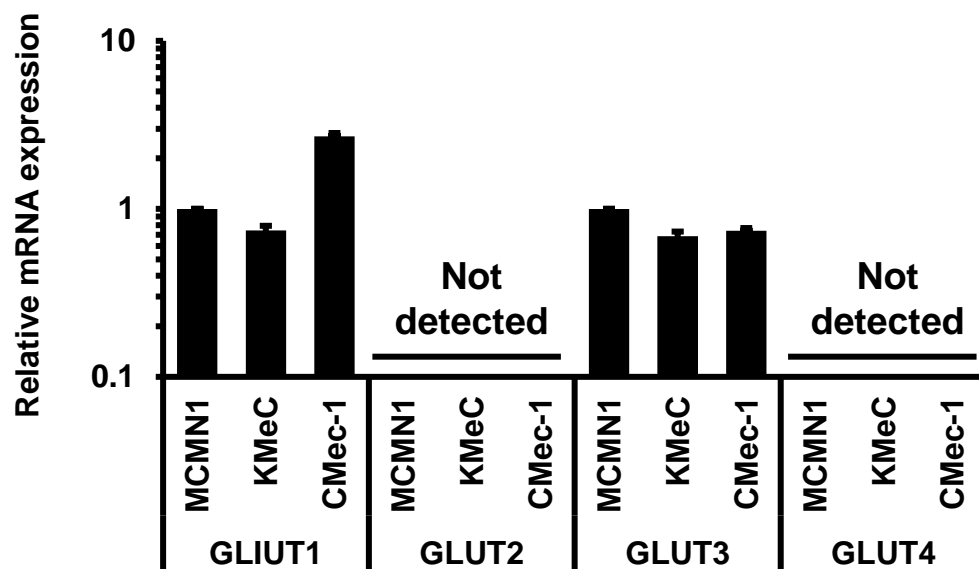

S6 Fig. The mRNA expression of GLUT isoforms in canine melanoma cell lines.

Supplement: S6 Fig — (PDF) [file pone.0243859.s006.pdf]

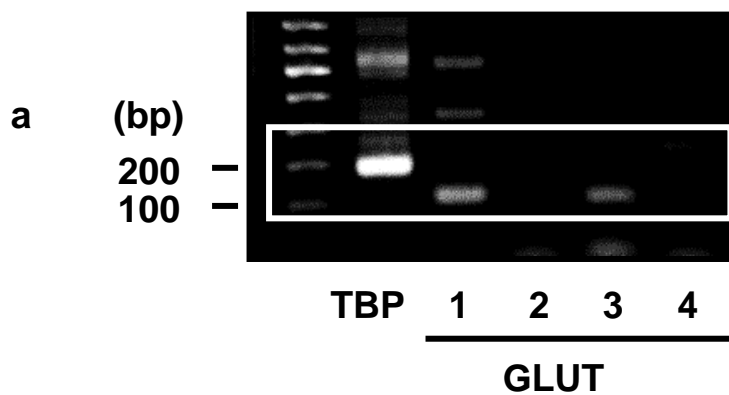

**b**

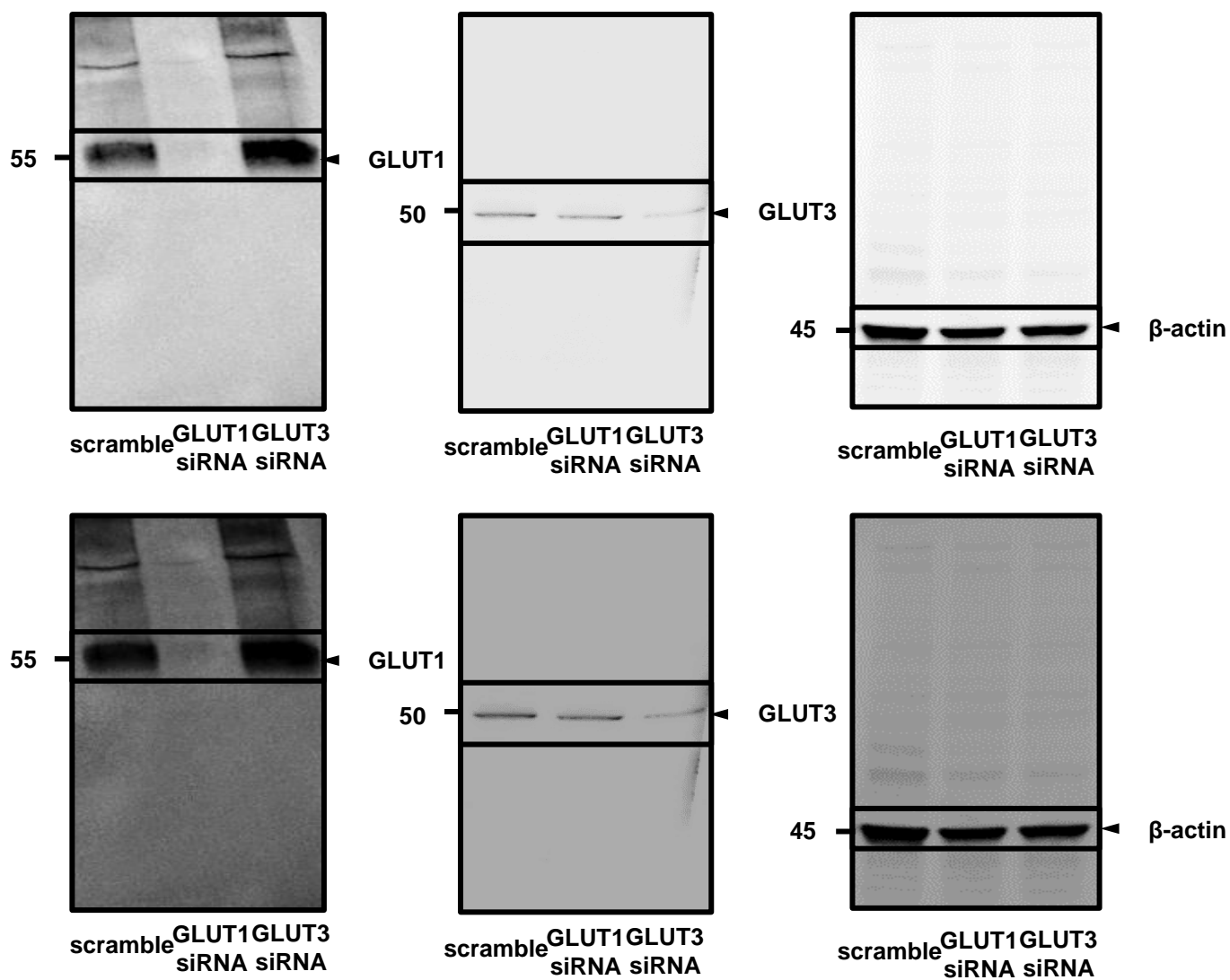

S7 Fig. Uncropped images for the blots shown in Fig. 3.

Supplement: S7 Fig — (PDF) [file pone.0243859.s007.pdf]
